# Activation Stoichiometry and Pore Architecture of TRPA1 Probed with Channel Concatemers

**DOI:** 10.1038/s41598-018-35435-y

**Published:** 2018-11-20

**Authors:** Wenlei Ye, Yu-Hsiang Tu, Alexander J. Cooper, Zheng Zhang, Vsevolod Katritch, Emily R. Liman

**Affiliations:** 1 Department of Biological Sciences, University of Southern California, Los Angeles, CA 90089 USA; 2 Present Address: Department of Physiology, University of California, San Francisco, CA 94158 USA; 3 Present Address: Zilkha Neurogenetics Institute, University of Southern California, Los Angeles, CA 90033 USA; 4 Department of Chemistry, University of Southern California, Los Angeles, CA 90089 USA

## Abstract

The nociceptor ion channel TRPA1 detects a wide range of hazardous chemicals, including reactive electrophiles such as cinnamaldehyde, which gate the channel allowing Na^+^ and Ca^2+^ entry. TRPA1 assembles as a tetramer, with a central pore within which an aspartate residue (D918) determines Ca^2+^ permeability. Here, we report that introduction of histidine at this position, D918H, makes TRPA1 channels sensitive to block by nanomolar concentration of Zn^2+^ and can be used to functionally tag subunits in concatemers. Concatemers with increasing numbers of D918H subunits display increasing sensitivity to Zn^2+^ inhibition, indicating that the four side chains at position 918 of the tetramer directly coordinate Zn^2+^ and other permeating divalent cations. In the published structure of TRPA1, this requires a rearrangement of the pore region which may represent the true open state of the channel. Concatemeric channels containing subunits mutated to be insensitive to reactive electrophiles (C622S) could be activated by cinnamaldehyde when as few as two subunits contained intact ligand binding sites. Activation upon liganding of just two of the four possible subunits may represent an optimal strategy to rapidly and reliably detect noxious chemicals.

## Introduction

Transient Receptor Potential A1 (TRPA1) is a non-selective cation channel that is activated by a wide range of irritants and is required for noxious sensations in animals^1,2^. Expressed in a subset of nociceptive afferent neurons, TRPA1 is activated by exogenous pungent chemicals in food or pollutants, such as allyl isothiocyanate (AITC, in wasabi), cinnamaldehyde (in cinnamon), acrolein (in motor vehicle exhausts) and permeating acids^3–6^. The activation of TRPA1 in afferent nerve endings depolarizes the membrane and generates warning signals to animals. TRPA1 can also be activated by endogenous inflammatory mediators such as bradykinin and trypsin through receptor-mediated mechanisms to cause hyperalgesia or itchy sensations^3,5,7,8^. Recent studies revealed that TRPA1 is also involved in many physiological or pathological processes in addition to sensory systems such as artery dilation and ischemia^9,10^. These studies illustrate a broad spectrum of TRPA1 functions^1,11^.

Similar to other TRP channels, a single TRPA1 channel subunit has six transmembrane helices with a pore-forming region between the 5^th^ and 6^th^ transmembrane domains^12,13^. One channel is composed of four subunits with their pore-forming regions facing the centre that make a single ion permeation pathway. Previous studies in our lab identified an aspartic acid residue at position 918 in rat TRPA1 (D918, homologous to human TRPA1 D915) neutralization of which dramatically reduces Ca^2+^ permeability^14^. D918 is also necessary for the permeation of other divalent ions such as Zn^2+^ ^15^. The cryo-electron microscopy (cryo-EM) structure of human TRPA1 revealed that D915 is located at the narrowest position of the channel, suggesting that the side chains at this position of the tetramer may comprise a portion of the selective filter^12^, but evidence that they directly interact with permeating divalent ions is still lacking.

The activators of TRPA1 include a broad range of chemicals without apparent structural similarities^1^, and which instead activate TRPA1 through their electrophilic properties^16,17^. These electrophiles bind covalently to cysteine residues within cytoplasmic regions of the channel, resulting in a conformational change that opens the channel. One critical cysteine residue is C622 in rodent TRPA1 (homologous to C621 in human TRPA1), located at the end of the ankyrin repeats, within the N-terminus of the protein^16–19^. The serine mutation of C622 (C622S) significantly reduces or completely eliminates the channel activation by electrophiles such as mustard oil and cinnamaldehyde, but activation by non-electrophilic compounds, such as by menthol and by polygodial, remains intact^20,21^. To function as a sensory receptor, TRPA1 would be expected to adopt a mechanistic strategy that allows it to detect electrophilic noxious chemicals rapidly and reliably. One hypothesis is that the covalent modification of a portion of the four C622 thiol groups in the channel tetramer should be sufficient to open the channel, such that no redundant modification is required. However, without a strategy to generate channels composed of wild type and C622S mutant subunits with a defined stoichiometry, the activation stoichiometry of the channel is not known.

Here, we generate a pore mutant of TRPA1, D918H, in which nanomolar concentrations of Zn^2+^ irreversibly inhibit the channel. We demonstrate that the high affinity Zn^2+^ inhibition requires the coordination of one Zn^2+^ ion with all four histidine side chains, implying that all four residues face the permeation pathway. Using concatemers containing a mix of wildtype and D918H mutant subunits, and probing with Zn^2+^, we establish the feasibility of generating channel tetramers with defined stoichiometries. By testing concatemers in which varying numbers of subunits carry the C622S mutation that renders them insensitive to activation by reactive electrophiles, we show that modification of two of the four C622 thiol groups in the channel tetramer is sufficient to open the channel.

## Results

### TRPA1 D918H is inhibited by Zn^2+^ and inhibition is reversed by acid wash

The pore region of TRPA1 contains an aspartate (D918 in the rat isoform and D915 in the human isoform) that when mutated to neutral residues renders the channel impermeable to divalent cations such as Ca^2+^ and Zn^2+^ ^14,15^. To determine whether the side chain of the residue at 918 directly coordinates permeating divalent ions or acts at a distance to electrostatically stabilize the permeating ions, we generated the mutant D918H which places a histidine residue at this position. Zn^2+^ interacts with the side chain imidazole of histidine and a high affinity binding site is created when multiple histidine residues coordinate a single Zn^2+^ ion^22,23^. To test the effect of this mutation, we measured whole-cell currents in HEK 293 cells transfected with either wild-type (WT) or D918H-mutant channels. TRPA1 currents were activated by exposure to the reactive electrophile cinnamaldehyde (Cin, 100 µM) in the absence of extracellular calcium to avoid inducing inactivation^14,24^. TRPA1 D918H channel was strongly activated by cinnamaldehyde, and like wild-type channels was sensitive to inhibition by the TRPA1 specific blocker, A-967079, consistent with A-967079 binding site being at the lower position of the permeating pathway away from D918^12^ (Fig. S1). Interestingly, another TRPA1 blocker, HC-030031^25^, was less effective at inhibiting D918H currents, indicating that HC-030031 may directly act on the pore (Fig. S1). Both wild-type and D918H currents were inhibited by extracellular acid (pH 4), although we did not compare the inhibition rates due to the contamination by acid-evoked endogenous Cl^−^ current in HEK cells (Fig. S1). These results show that D918H makes a functional ion channel that can be activated by reactive electrophiles.

**Figure 1:**
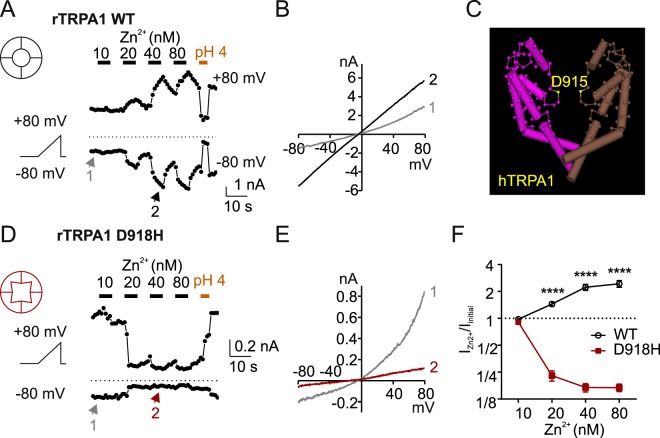
Histidine substitution at D918 in TRPA1 generates a high affinity Zn^2+^ inhibitory site. (**A**,**D**) Effect of extracellular Zn^2+^ on TRPA1 currents in HEK 293 cells transfected with WT or D918H TRPA1 channels. TRPA1 current was elicited by exposure to cinnamaldehyde (100 µM). The membrane potential was held at −80 mV and ramped (−80 mV to +80 mV, 1 V/s) once every second. (**B**,**E**) The I-V relationships of currents shown at indicated time points. Zn^2+^ potentiates the WT current and blocks D918H currents. (**C**) The structure of human TRPA1 modified from published data on National Center for Biotechnology Information (NCBI), showing a side view of the S5~S6 transmembrane domains from two opposing subunits in a TRPA1 channel. The backbones of D915 residues (homologous with rat TRPA1 D918) are highlighted. (**F**) Average data from experiments as in A,D showing the magnitude of the currents in the presence of Zn^2+^, at the concentration indicated, relative to the starting current level I_Zn2+_/I_initial_ (measured at +80 mV). ****P < 0.0001 (Sidak’s multiple comparison following two-way ANOVA); *N* ≥ 4.

We next tested the sensitivity of wild type and D918H channels to Zn^2+^. As previously reported, extracellular application of Zn^2+^ reversibly potentiates wild-type TRPA1 due to entry of Zn^2+^ through the channel^15^ (Fig. 1A,B). In contrast, we found that nanomolar concentration of Zn^2+^ inhibited D918H (Fig. 1D,E), with near complete inhibition of the currents by 20 nM Zn^2+^ (77.1 ± 3.0%). The currents did not recover from inhibition during a 6-second wash-off. Interestingly the block was rapidly reversed by exposure to a strongly acidic solution (pH 4), suggesting that H^+^ is able to protonate H918, even when bound to Zn^2+^, thereby reducing its affinity for Zn^2+^ (Fig. 1D,E). Full recovery was observed following removal of the acidic solution, which itself blocks the channel^6^. To explore this possibility in more detail, we extended the duration of the exposure to each concentration of Zn^2+^. Acid wash was applied before each application of Zn^2+^ to restore the currents to its initial magnitude. As shown in Fig. S2, a 60-second exposure allows an even lower concentration of Zn^2+^ (5 nM) to block a large fraction of the D918H currents, and even after 60 seconds the decay of the currents had not reached equilibrium. Fitting the decay of the current amplitude with a single-exponential equation shows that the time constant of inhibition is dependent on Zn^2+^ concentration. These data indicate that the Zn^2+^ inhibition of TRPA1 D918H is essentially irreversible at neutral pH. This result can best be explained if the side chains of amino acid residues at position 918 face the permeation pathway of the channel.

### A concatemer of wild type and D918H displays an intermediate phenotype

The structure of TRPA1 revealed by cryo-EM shows that D915 of human TRPA1 is located at the narrowest point of the channel^12^. The strong inhibition of D918H by Zn^2+^ implies that the side chains of the amino acid at this position directly interact with the permeating divalent ions. To test whether sensitivity to Zn^2+^ depends on the number of histidine residues at this position in the pore, we generated a concatemer composed of one wild-type subunit and one D918H subunit (WT-D918H), which is expected to yield channels with 2 wild-type subunits and 2 D918H subunits. In cells transfected with WT-D918H, large whole-cell currents were elicited in response to cinnamaldehyde, which were strongly inhibited by the TRPA1 blocker A-967079 and only partially blocked by HC-030031 (Fig. S3).

**Figure 2:**
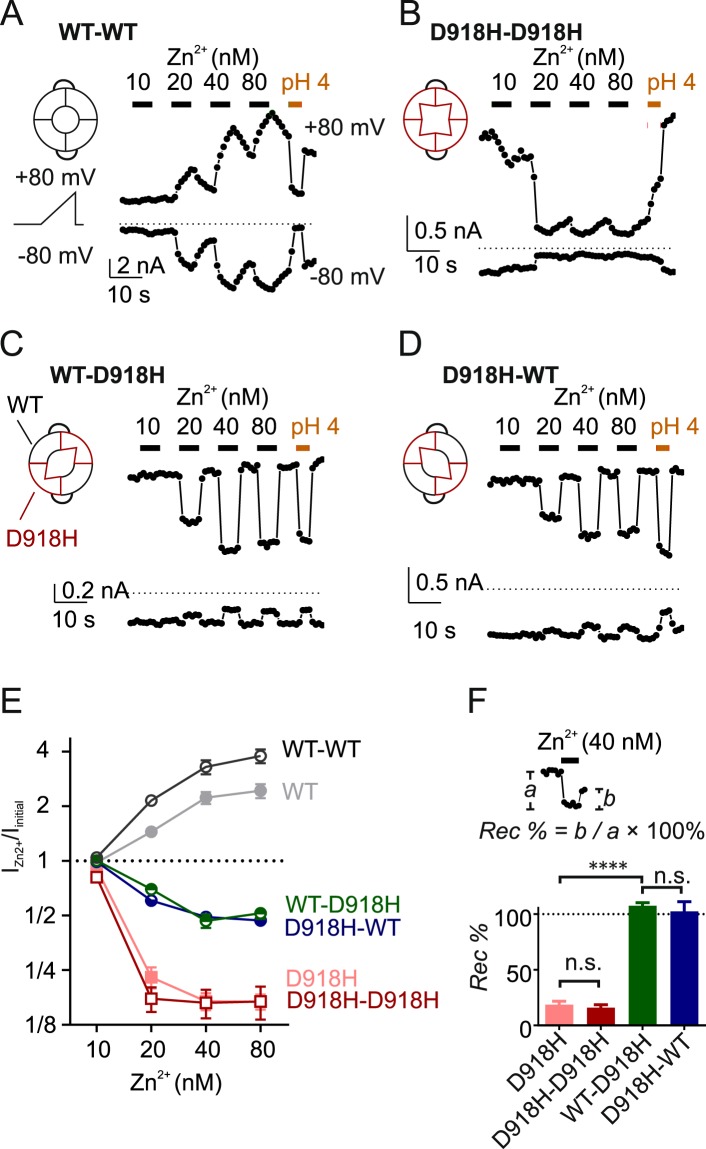
Concatemers consisting of WT and D918H subunits display an intermediate Zn^2+^-sensitivity. (**A**–**D**) Effect of Zn^2+^, at the time and dose indicated, on currents from TRPA1 dimers composed of the four permutations of WT and D918H subunits. Currents were activated by pretreatment with cinnamaldehyde (100 µM) and were measured at +80 mV and −80 mV from ramp depolarizations applied once per second. (**E**) Average data from experiments as in A-D showing the magnitude of the currents in the presence of Zn^2+^, at the concentration indicated, relative to the starting current level I_Zn2+_/I_initial_ (measured at +80 mV). Data from WT and D918H monomers were replotted from Fig. 1F. (**F**) Recovery percentage (*Rec %*) of the currents from inhibition following removal of Zn^2+^ (40 nM) was quantitated as shown. n.s. *P* > 0.05, ****P < 0.0001 (Tukey’s multiple comparison following one-way ANOVA); *N* = 5.

We next tested the sensitivity to Zn^2+^ of the WT-D918H channel as well as WT-WT and D918H-D918H channels. The WT-WT and D918H-D918H channels had the same Zn^2+^ sensitivity as WT and D918H monomers, respectively, ruling out that construction of concatemers itself affected the pharmacology of the channels (Fig. 2A,B). In contrast, the WT-D918H currents displayed a distinct sensitivity to Zn^2+^, unlike that of either WT or D918H alone. Most strikingly, Zn^2+^ inhibited the WT-D918H currents reversibly at the same concentration of Zn^2+^ that irreversibly blocked D918H alone and produced no potentiation, in contrast to its effect on WT channels (Fig. 2C,E,F). Reversing the order of the subunits to generate the concatemer of D918H-WT did not affect the Zn^2+^ sensitivity of the currents, indicating that the phenotype was determined by the composition and not the order of the subunits (Fig. 2D–F). To confirm that the distinct response of the concatemer is not due to random association of wild-type and D918H subunits, we co-transfected the cells with wild-type (fused with YFP) and D918H (fused with tag-RFP) TRPA1 vectors in various ratios and measured the Zn^2+^ sensitivity of doubly-transfected cells. These doubly-transfected cells showed a variety of phenotypes, including currents that appeared to be dominated by wild-type channels and those dominated by D918H channels, depending on the co-transfection ratios (Fig. S4). However, in cells that showed an intermediate phenotype, the responses were qualitatively different from the responses of the linked WT-D918H channels. Notably, in the co-transfected cells, Zn^2+^ produced biphasic responses in which the currents were initially blocked and then potentiated indicative of the presence of homotetrameric WT channels (Fig. S4B).

**Figure 3:**
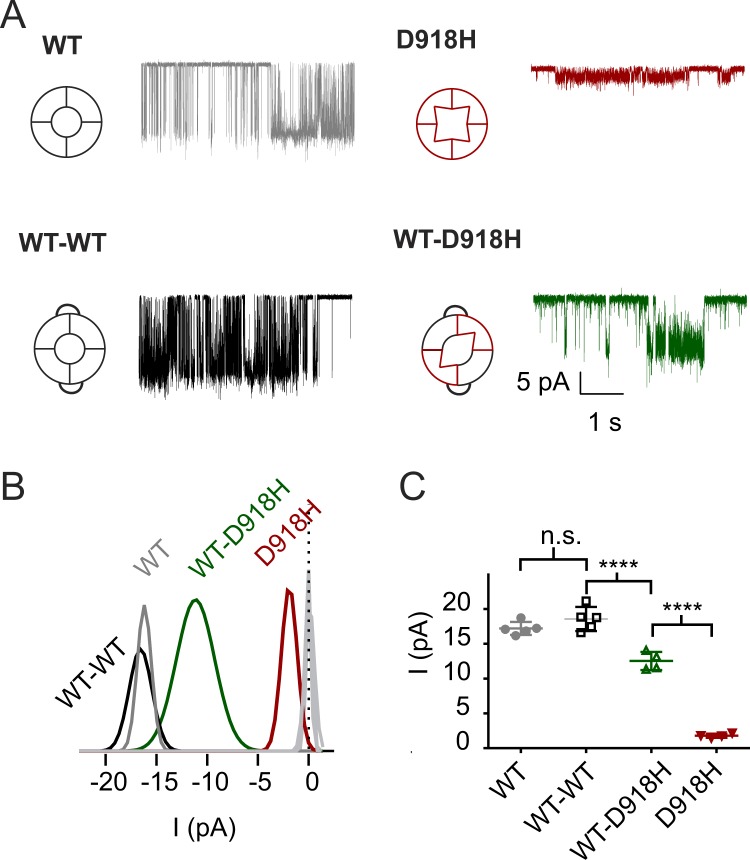
WT-D918H dimers display an intermediate single-channel conductance. (**A**) The unitary currents of WT, D918H, and WT-WT, WT-D918H channels recorded with cell-attached configuration. The bath solution was K^+^-based to zero cell membrane potential, and the current was recorded at −80 mV. (**B**) All points histograms fitted with Gaussians indicate that the WT-D918H displays an intermediate single channel conductance. (**C**) Scatter plot showing the single channel conductance of the indicated channels. n.s. *P* > 0.05, ****P < 0.0001 (Tukey’s multiple comparison following one-way ANOVA).

If the concatemers assembled with a defined stoichiometry, we expected this to be reflected at the single channel level. To test this possibility, we measured the single channel conductance of WT, WT-WT, D918H, and WT-D918H channels following activation by cinnamaldehyde in cell-attached patches from transfected cells. D918H channels had much smaller single channel currents than WT channels (D918H, 1.8 ± 0.3 pA versus WT, 17.2 ± 0.9 pA at −80 mV) while current amplitudes of WT-WT channels were no different than those of WT channels (Fig. 3). WT-D918H channels showed an intermediate current amplitude of 12.5 ± 1.3 pA. Importantly, we did not observe a heterogenous population of conductances for the concatemer, as predicted if the subunit stoichiometry was not constrained (Fig. 3). Together these results indicate that the concatemer assembles to make tetrameric channels with a defined subunit stoichiometry.

**Figure 4:**
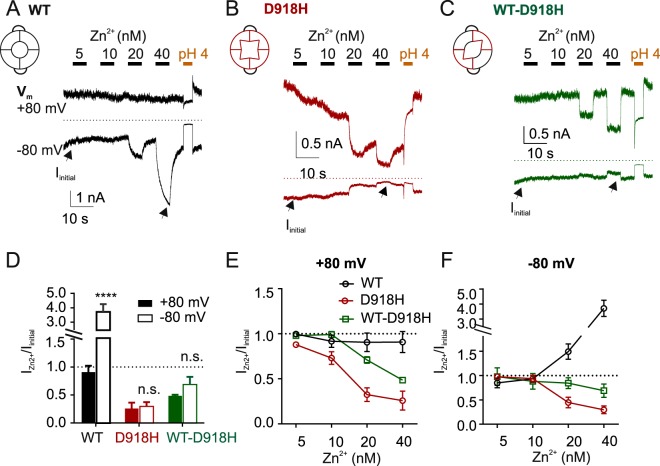
The block of D918H and WT-DH does not require the permeation of Zn^2+^. (**A**–**C**): Effect of Zn^2+^ on currents from TRPA1 dimers measured with a stable holding potential of either +80 mV or −80 mV throughout the duration of the solution exchange. Traces were recorded sequentially but are shown on the same axes for illustrative purposes. Currents were activated by pretreatment with cinnamaldehyde (100 µM). (**D**) Summary of fraction change of TRPA1 WT, D918H and WT-D918H currents in response to Zn^2+^ (40 nM). I_Zn2+_/I_initial_ was calculated from the current magnitude measured 5 seconds after application of Zn^2+^, relative to that before Zn^2+^ application. n.s. *P* > 0.05, ****P < 0.0001 (Sidak’s multiple comparison following two-way ANOVA); *N* ≥ 4. (**E**,**F**): Average data from experiments as in A-C showing the magnitude of the currents in the presence of Zn^2+^, at the concentration indicated, relative to the starting current level I_Zn2+_/I_initial_ with a holding potential of +80 mV (**E**) or −80 mV (**F**). Note that while the potentiation of WT channels by Zn^2+^ is very sensitive to membrane potential, the inhibition of the D918H and WT-D918H channels is not very sensitive to membrane potential.

To further characterize the WT-D918H concatemer, we examined the voltage-dependence of Zn^2+^ inhibition by holding the voltage at either −80 mV or +80 mV throughout the duration of the experiment. Note that in the previous experiments (Figs 1 and 2), voltage ramps were applied from −80 to +80 mV to allow us to record both inward and outward currents, but because equilibrium was not reached, these experiments could not be used to measure the voltage-dependence of Zn^2+^ inhibition. Holding at +80 mV prevented potentiation of the WT channel by Zn^2+^, which requires Zn^2+^ entry^15^, while strong potentiation was observed when the cells were held at −80 mV (Fig. 4A). In contrast, Zn^2+^ inhibited both D918H and WT-D918H regardless of the holding potentials, with little evidence of any effect of voltage on the sensitivity to Zn^2+^. (Fig. 4B~F). These results indicate that Zn^2+^ inhibition of D918H and WT-D918H does not require the permeation of Zn^2+^ through the channel. Moreover, they indicate that the binding site for Zn^2+^ in both channels is located near the outer surface of the ion conduction pathway and not deep within the membrane electrical field.

Together these results demonstrate that a concatemer of TRPA1 subunits can be generated that constrains the subunit stoichiometry. Inclusion of the D918H subunit in the concatemer generates a channel with distinct pharmacological and single channel properties thus providing a method for functionally tagging subunits.

### Rate of Zn^2+^-inhibition depends on the number of histidine residues in the pore

In order to determine if all four side-chains at position 918 contribute to the interaction with permeating ions, we generated concatemers of four TRPA1 subunits in which between one and three subunits harbored the D918H mutation. For these experiments, the unlike subunit was placed in the last position (e.g. WT-WT-WT-D918H and D918H-D918H-D918H-WT), to allow us to confirm whether the subunit in this position had been incorporated in the channel. We first measured ion selectivity, which was expected to vary among the channels which carry mutations in a residue critical for divalent cation permeability. As expected, the Ca^2+^ selectivity progressively decreased with the reduction in the number of wild-type subunits in the channel, while selectivities for Na^+^ and K^+^ were not altered as compared with Cs^+^ (Fig. S5). These results show that four aspartic acid residues are necessary at position 918 for maximal Ca^2+^ selectivity of TRPA1.

**Figure 5:**
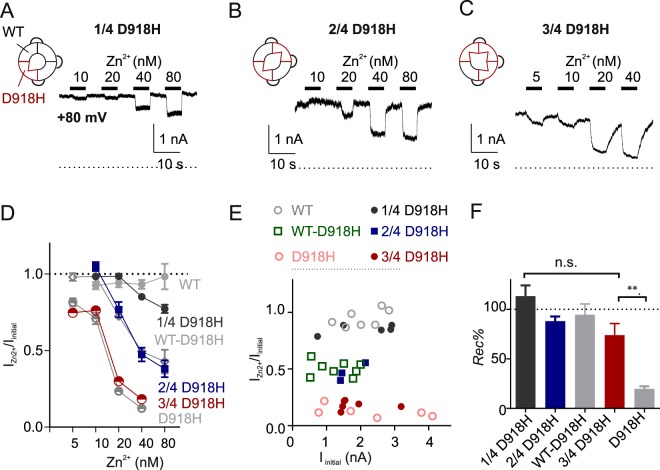
The Zn^2+^ sensitivity progressively increases with the number of D918H subunits in the TRPA1 tetramer. (**A**~**C**) Time courses of Zn^2+^ responses of TRPA1 concatemers composed of 4 TRPA1 subunits. The currents were activated by pretreatment with cinnamaldehyde (100 µM) and were all recorded with holding at +80 mV to prevent any Zn^2+^ entry. (**D**) Summary of current fraction changes of the tetramers under indicated Zn^2+^ conditions. (**E**) The scatter plot of current fraction changes in 40 nM Zn^2+^ against the current magnitudes. The data were replotted from Figs 4E and 5D, showing the inhibition rate does not correlate with current magnitude. (**F**) Summary of the recovery percentage (*Rec %*) at the wash-off of Zn^2+^ (40 nM). n.s. *P* > 0.05, **P < 0.01 (Tukey’s multiple comparison following one-way ANOVA); *N* ≥ 3.

We then tested if tetrameric concatemers with different number of histidine residues in the pore have distinct Zn^2+^ affinities. For these experiments, we generated concatemers containing 1–3 D918H subunits and tested their sensitivities to Zn^2+^. Note that inhibition was measured at +80 mV, to avoid permeation of Zn^2+^ and consequent potentiation which might confound our results. As shown in Fig. 5A–D, the Zn^2+^ inhibition of the currents was increasingly more potent as the number of D918H subunits in the concatemer increased, with each concatemer showing a distinct pharmacological profile. Notably, block of channels containing 1-2 D918H subunits was rapidly reversible. Recovery of channels containing 3 D918H subunits from Zn^2+^ inhibition was slower but nonetheless distinguishable from otherwise irreversible inhibition of D918H alone in spite of a similar amount of block (Fig. 5F). Together these data indicate that only the tetramer with four D918H subunits (i.e. D918H alone) generates a channel that binds Zn^2+^ irreversibly.

### Covalent modification of C622 in two subunits in the channel tetramer is sufficient to open the channel

The above experiments indicate that we can generate concatemers of TRPA1 subunits with defined stoichiometries that can be verified by tagging subunits with D918H mutation. We reasoned that these constructs could be used to address questions regarding the activation stoichiometry of TRPA1. In particular, we wondered how many subunits of TRPA1 must be covalently modified by reactive electrophiles for the channel to open.

**Figure 6:**
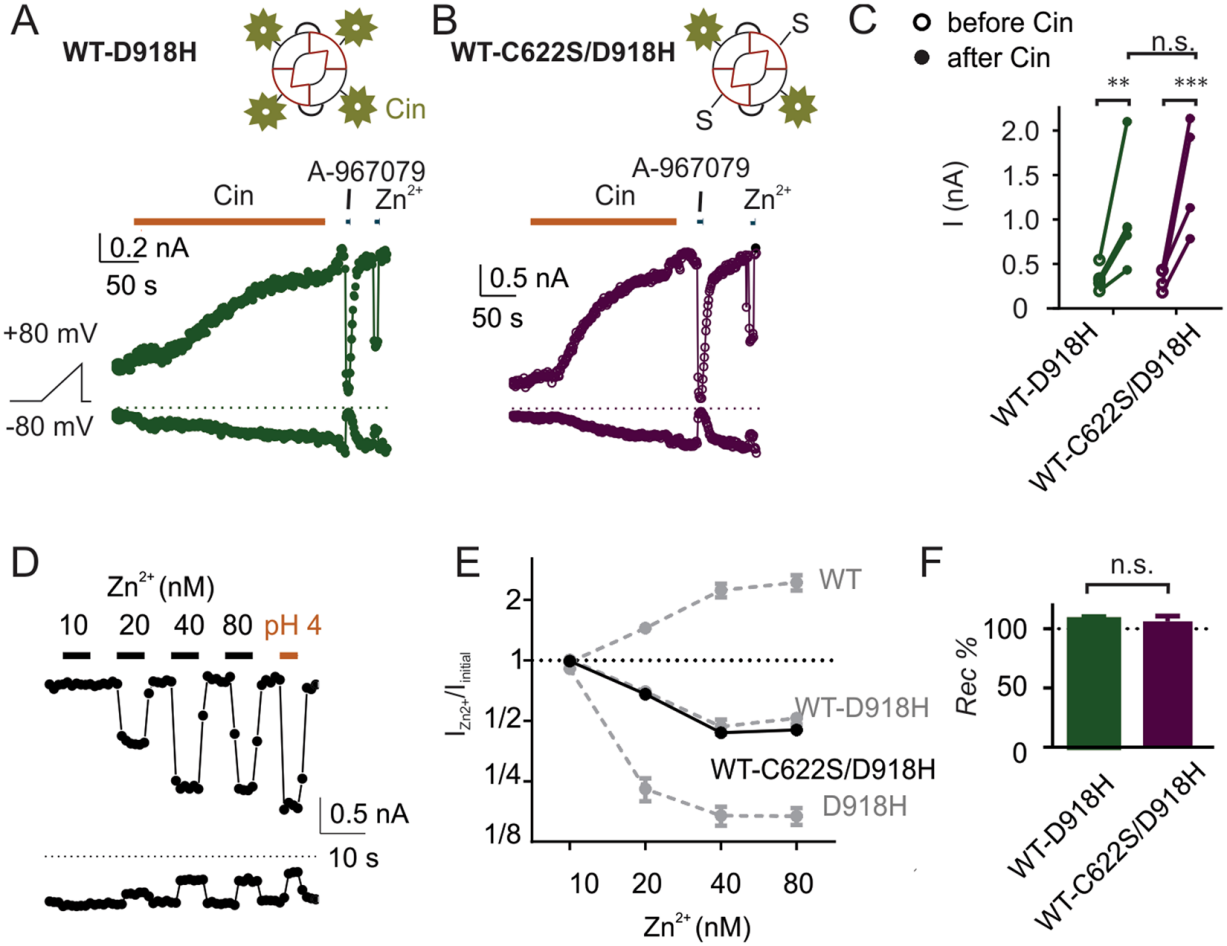
TRPA1 channels with 2 subunits harboring the C622S mutation can be activated by reactive electrophiles. (**A**,**B**) Activation by 100 µM cinnamaldehyde of currents in cells transfected with WT-D918H or WT-C622S/D918H. In both cases, cinnamaldehyde activated a current that could be inhibited by the TRPA1-specific blocker, A967079, and by Zn^2+^ (40 nM), reversibly. (**C**) Summary of current magnitudes measured before and after cinnamaldehyde treatment from experiments as in (**A**,**B**). **P < 0.01, ***P < 0.001 (Tukey’s multiple comparison following two-way ANOVA). (**D**) Inhibition of WT-C622S/D918H currents by the indicated concentrations of extracellular Zn^2+^. A pH 4 solution was used as a control; inhibiton of currents was qualitatively similar to that of WT-D918H currents. (**E**) Dose-dependence of inhibition by Zn^2+^ responses of WT-C622S/D918H currents (*N* = 5) is similar to that of WT-D918H. Data for WT, WT-D918H and D918H currents were replotted from Fig. 2. (**F**) Summary of recovery percentage (*Rec %*) at the wash-off of 40 nM Zn^2+^. n.s. *P* > 0.05 (unpaired *t*-test); *N* = 5.

To address this question, we generated concatemers of wild-type subunits and subunits bearing C622S mutation, which was previously shown to essentially eliminate electrophile activation^16–19^. To test whether all four subunits need to be liganded to open the channel, we generated the concatemer WT-C622S/D918H, in which the second subunit harbors both the cysteine mutation and the pore mutation. Note that the presence of the pore mutation in the second subunit allows us to confirm that the second subunit is incorporated in the channel, using the distinct concatemer pharmacology described above. Strikingly, the WT-C622S/D918H concatemer was strongly activated by cinnamaldehyde (100 µM) and the magnitudes of the currents were similar to those for dimeric channels with all four cysteine intact (i.e. WT-D918H, Fig. 6A–C). We also tested whether WT-C622S/D918H concatemers could be activated by polygodial (PLGD), a non-electrophile TRPA1 agonist that is capable of activating the mutant C622S (Fig. S6A,B). We observed no significant difference in the magnitude of the currents activated in cells expressing WT-C622S/D918H or WT-D918H, indicating that the two channel constructs expressed at similar levels (Fig. S6C–E). Importantly, cinnamaldehyde- and PLGD-activated WT-C622S/D918H currents were reversibly inhibited by Zn^2+^ and the sensitivity to Zn^2+^ of the cinnamaldehyde-activated currents was indistinguishable from that of the WT-D918H currents (Figs 6D–F, S6F). These results demonstrate that the second subunit, C622S/D918H, is incorporated in the channel and that elimination of the cysteines at 622 in two of the four subunits that compose the channel does not disrupt the channel activation by the reactive electrophile, cinnamaldehyde.

**Figure 7:**
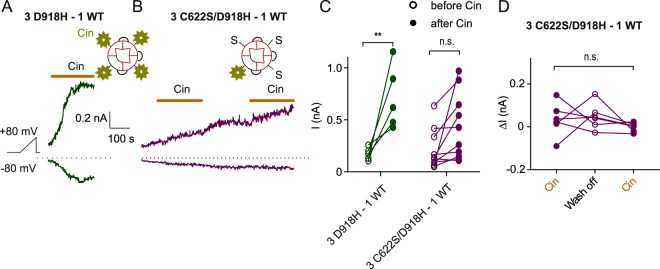
TRPA1 channels with 3 subunits harboring C622S mutation cannot be activated by cinnamaldehyde. (**A**) Response to 100 µM cinnamaldehyde of currents in cells transfected with 3 D918H - 1 WT and 3 C622S/D918H - 1 WT. Note that in (**B**), the current is spontaneously increasing and there is no correlation with the application of cinnamaldehyde. (**C**) Summary of current magnitudes measured before and after treatment of cinnamaldehyde. **P < 0.01 (Sidak’s multiple comparison following two-way ANOVA). (**D**) Summary of current magnitude changes during a 100 s window with or without cinnamaldehyde. n.s. *P* > 0.05 (repeated measures one-way ANOVA).

We next asked whether activation by reactive electrophiles of a single subunit is sufficient to open the channel. We generated the concatemer of three C622S/D918H subunits fused with a wild type subunit (3 C622S/D918H – 1 WT), and compared this channel with concatemers containing the same pore mutations and four intact cysteines (3 D918H – 1 WT). For the concatemer with one functional cysteine (3 C622S/D918H – 1 WT), we failed to observe an increase in the currents correlating with the application of cinnamaldehyde (Fig. 7), notwithstanding that some cells spontaneously developed currents independently of cinnamaldehyde application (Fig. 7B,D). These results suggest that one subunit with C622 is not sufficient for normal activation of TRPA1 by reactive electrophiles.

Together our results demonstrate that, in the tetrameric channel, the presence of two subunits bearing cysteine at position 622 is sufficient to allow electrophiles to activate the channel, while one subunit is not enough.

## Discussion

**Figure 8:**
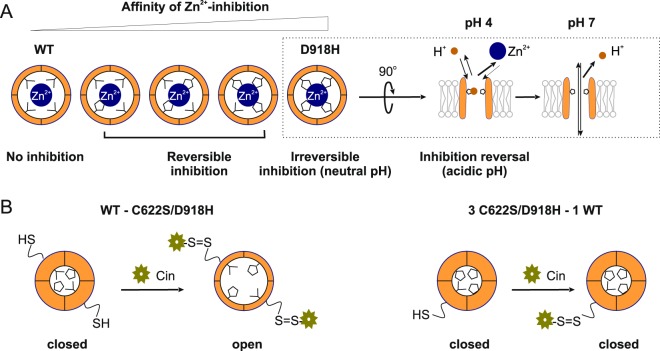
Summary. (**A**) Channel tetramers containing increasing numbers of histidine residues at position 918 (the narrowest point in the permeation pathway) display increasing sensitivities to Zn^2+^-inhibition. For channels containing 4 D918H subunits, Zn^2+^-inhibition is essentially irreversible at neutral pH. At low pH. protons displace Zn^2+^, and block the channel; subsequent wash-off of acid reverses the inhibition. See data in Figs 1~5. (**B**) The dimer WT-C622S/D918H is activated by cinnamaldehyde. The Zn^2+^-responses show the pore of WT-C622S/D918H is identical to that of WT-D918H, verifying the inclusion of subunits harboring the C622S mutation. In contrast, tetramers containing just one subunit that can be covalently modified by cinnamaldehyde are not activated. See data in Figs 6 and 7.

The structure of TRPA1 revealed by cryo-EM shows that D918 is positioned at the narrowest point of the permeation pathway, consistent with previous electrophysiological studies^12,14^. Here, we generated a D918H mutant and provide evidence that the side chains of all four H918 residues of the tetramer coordinate one permeating divalent cation (Fig. 8A), which might represent the mechanism of Ca^2+^ selectivity in wild-type TRPA1 channels. We show that concatemers that contain varying numbers of wild-type and D918H mutant channels show distinct sensitivities to Zn^2+^, allowing us to generate channel tetramers with defined and verifiable stoichiometries. Using concatemers in which some of the subunits carry the C622S mutation, rendering them insensitive to reactive electrophiles, we found that the covalent modification of C622 residues on two subunits is sufficient to open the channel (Fig. 8B). Our study thus extends our understanding of the TRPA1 pore architecture and activation stoichiometry.

**Figure 9:**
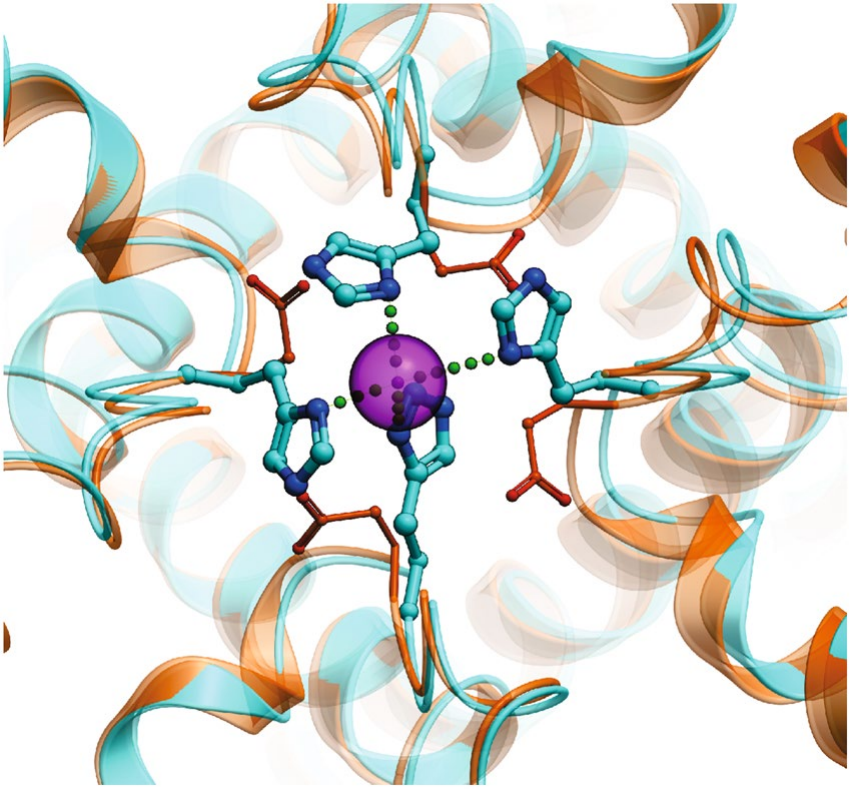
Model of the Asp915His mutant based on the human TRPA1 electron cyro-microscopy structure (PDB ID: 3J9P). Zn^2+^ ion shown in magenta, the original electron microscopy structure colored orange, the optimized model of the mutant colored cyan. Coordination of Zn^2+^ shown by green dotted lines.

Differences in ion selectivity among TRP channels can be attributed to their unique pore architectures. The high selectivity for Ca^2+^ over monovalent ions in many cases correlates with the presence of acidic amino acids in the narrowest part of the pore (selectivity filter), potentially coordinating the permeating Ca^2+^ ^13^. Among the TRPV channels with available high-resolution structures, TRPV6 has a P_Ca2+_/P_Na+_ of >100 while TPRV1 has a P_Ca2+_/P_Na+_ of 1~5^26,27^; this may be explained by the presence in TRPV6 of three Ca^2+^ coordination sites lined up as a funnel with the first one, D541, highly favoring Ca^2+^, while the narrowest point in TRPV1 is formed by the backbone carboxyl oxygen of glycine and the side chain of methionine^28,29^. The selectivity of TRPA1 for Ca^2+^, however, is intermediate between those of TRPV1 and TRPV6 (P_Ca2+_/P_Na+_  = 5.7~7.9)^30^, and previous studies have shown that the negative charge of D918 is crucial for Ca^2+^ selectivity^14^. Our results suggest that the four side chains of D918 in the tetramer directly coordinate a single permeating divalent cation, which may explain why TRPA1 is selective for Ca^2+^ over monovalent cations. In the structure of TRPA1, this could be accomplished with a rearrangement of the pore region with four His918 side chains coordinating one Zn^2+^ ion, and this modified conformation may represent the true open state of the channel. The model of H915 tetramer is shown in Fig. 9, as compared to the WT structure of TRPA1. Our conformational modeling indeed suggests that optimal positioning of the H915 side chains for the preferred tetrahedral Zn^2+^ coordination^31^ requires some substantial conformational changes in the loop that open up the pore.

Our data suggest different mechanisms of action for the two specific TRPA1 blockers, HC-030031 and A-967079. A-967079 binds to the site in the vestibule of the channel pore down the ion-conducting pathway^12^. It interacts with the transmembrane helices 5 and 6, induces a conformational change that stabilizes the binding and thus inhibits the ion permeation. The position of A-967079 binding site is at a distance along the pore funnel from D918, so it is not surprising that mutation of D918 does not interfere with the inhibition by this antagonist. The electron density of HC-030031 was not resolved in the TRPA1 cryo-EM. Our results show that HC-030031 does not block D918H or WT-D918H, indicating that it may bind in the pore, and be stabilized by the presence of acidic residues.

TRPA1 has a multiphasic response to agonists, reflecting a possible presence of multiple open states. A prolonged exposure to agonists has been proposed to trigger pore dilation, in which the current-voltage relationship changes from outwardly rectifying to linear and the channel increases its permeability to large-sized monovalent ions such as tetramethylammonium (TMA^+^) and N-methyl-D-glucamine (NMDG^+^)^32^. The elevation of intracellular Ca^2+^ or Zn^2+^ concentration also potentiates the currents and linearizes the current-voltage relationships^14,15^ although whether these changes affect the pore architecture is not known. Moreover, the TRPA1 channel open conformation is not stable in that further elevation of and/or prolonged exposure to high intracellular Ca^2+^ induces channel inactivation^14,24^. These facts might make it difficult to obtain TRPA1 channels in the open conformation in samples prepared for structural studies. Our study provides a means to generate a TRPA1 mutant which could be used to trap the channels in the open state for structural studies.

The stoichiometry of ligand-activation has been examined in the thermosensitive TRP channel, TRPV1 and TRPM8. For TRPM8, liganding of each subunit by menthol shifts the voltage-dependence of activation, such that the maximal activation is observed only when all four subunits are liganded^33^. Similarly, activation of TRPV1 by protons can be observed even when only one subunit contains the titratable residues E600 and E648 required for proton activation, but maximal activation is observed only when all four subunits contain the titratable residues^34^. In contrast, activation of TRPV1 by capsaicin is more “tolerant” and binding of capsaicin to a single TRPV1 subunit is sufficient to fully open the channel^34^. The presence of four ligand binding sites increases the sensitivity of the channel to capsaicin, but does not change the maximal response. We find that the activation stoichiometry of TRPA1 is somewhere between these extremes, with liganding of two subunits at C622 sufficient to activate the channel, while liganding of one subunit is not. This is consistent with data from calcium imaging, which shows a Hill coefficient for TRPA1 activation by Δ9-tetrahydrocannabinol and cinnamaldehyde of around two, although these data need to be interpreted with caution due to the fact that the agonists are irreversible and that calcium imaging is a very indirect measure of channel activity^4,35^. Interestingly, two is also the number of ligands necessary to open the structurally unrelated pentameric neurotransmitter receptors, such as the α4β2 nicotinic acetylcholine receptor^36^. Liganding of two subunits may allow TRPA1 to strike an optimal balance between speed and reliability, not requiring redundant signals and meanwhile preventing pain inducing “false alarms”.

Our study shows that tetrameric TRPA1 channels can be generated with defined and verifiable subunit compositions and it provides evidence that liganding of two subunits of TRPA1 at C622 is sufficient to open the channel. It also raises further questions regarding the detailed mechanism of channel activation. When one subunit of the tetramer binds to an activator molecule, how does it pass the message on to the other unbound partners within the tetrameric channel? Also, not known is whether the activation stoichiometry is similar for non-electrophilic activators such as polygodial, menthol and divalent cations. Detailed understanding of the mechanism of activation of TRPA1 may allow the manipulation of its response to subsets of activators and provide clues to guide the development of pharmaceuticals to treat pain and other pathological processes^1,11^.

## Materials and Methods

### cDNAs and heterologous expression

The N-terminal YFP fusion of rat TRPA1 (YFP-TRPA1) was as previously described^14^. Point mutations were generated by QuikChange mutagenesis (Stratagene, La Jolla, CA) and were verified by sequencing (Retrogen, San Diego, CA). TagRFP-fused TRPA1 D918H was generated by replacement of YFP with tag-RFP using the infusion reagent (Takara Bio, Mountain View, CA).

For tandem TRPA1 constructs, we designed constructs containing cDNAs in this order: 5′ YFP – Linker 1 – TRPA1 subunit 1 (S1) – Linker 2 – TRPA1 subunit 2 (S2), in pcDNA3. The sequence for Linker 1 is ATACCGGTCAACTTTGGCAGATCGCGAGCCACCGCTAGCACC (IPVNFGRSRATAST in amino acid sequence), containing an *Nhe*I cut site (underlined). The sequence of Linker 2 is GTTTTGCTTAAG (VLLK in amino acid sequence), containing an *Afl*II cut site (underlined). For TRPA1 tetramers, the plasmids were designed as: 5′ YFP – Linker 1 – TRPA1 subunit 1 – Linker 2 – TRPA1 subunit 2 – Linker 3 – TRPA1 subunit 3 – Linker 4 – TRPA1 subunit 4, in pcDNA3. The sequence of Linker 1 is ATACCGGTGGGTTCAGCCCGCGGA (IPVGSARG, amino acid sequence), with *Age*I cut site (underlined); Linker 2 is GGATCTGCAGGGCGCGCC (GSAGRA), with *Asc*I cut site; Linker 3 is GGCTCAGCTGCTAGCACC (GSAAST), with *Nhe*I cut site; Linker 4 is GTTTTGCTTAAG (VLLK), with *Afl*II cut site (Fig. S7). Bacteria transformed with vectors of 4-subunit concatemers needed to be grown at 28 °C. Sequences of all constructs were validated by restriction enzyme digestion and Sanger sequencing (Integrated DNA Technologies, Skokie, IL). Sequence of each subunit was obtained following PCR amplification using linker-specific primers.

HEK 293 cells were recorded 24 hours after transfection with TransIT®-LT1 (Mirus Bio Corp., Madison, WI) of 500 ng plasmid/well (six-well chamber) in most experiments. Cells in experiments as shown in Figs 6 and S6 were transfected with 200 ng plasmid/well. Cells in experiments in Fig. 7 were transfected with 100 ng plasmid/well. Prior to recording, cells were treated with 0.05% trypsin for 2 min at 37 °C and replated in the recording chamber in Tyrode’s solution. Transfected cells were identified under epifluorescence. Recordings were performed at room temperature within 6 hours after cells were lifted.

### Patch Clamp Recording

Patch-clamp electrophysiology was performed as previously described^14^. In brief, cells were perfused with a Ca^2+^-free solutions following formation of a gigaohm seal. Cells were lifted and moved in front of a linear array of microperfusion pipes (Warner Instruments, Hamden, CT) during recording. Currents were recorded with an Axopatch 200B amplifier, sampled at 5 kHz, and filtered at 1 kHz. Data were digitized with a Digidata 1322a digitizer, acquired with pClamp 9 (Axon Instruments, Union City, CA) and analyzed with GraphPad Prism 6 (Graphpad, La Jolla, CA). Representative data shown in figures were exported into Origin (Microcal, Northampton, MA) and CorelDRAW (Corel Corp., Eden Prairie, MN). For most whole-cell recordings the membrane potential was held at −80 mV and ramped from −80 mV to +80 mV (1 V/s) once every second, unless otherwise stated. For cell-attached recordings, cells were constantly held at −80 mV.

### Solutions

Tyrode’s solution contained (mM): 145 NaCl, 5 KCl, 1 MgCl_2_, 2 CaCl_2_, 20 dextrose, 10 HEPES (pH 7.4 with NaOH). Divalent-free bath solution was as follows (mM): 150 NaCl, 10 HEPES, 0.5 EGTA or 5 EGTA. In pH 4 solution, HEPES was replaced with MES. Zn^2+^-containing solutions were obtained by adding 0.396, 0.442, 0.469, 0.484, 0.492 Zn^2+^ (mM) to a solution containing 0.5 mM EGTA to obtain free Zn^2+^ concentrations of 5 nM, 10 nM, 20 nM, 40 nM, 80 nM respectively (For Figs 1, 2, S4), or by adding Zn^2+^ (3.96, 4.42, 4.69, 4.84, 4.92 mM) to a solution containing 5 EGTA to obtain free Zn^2+^ concentrations of 5 nM, 10 nM, 20 nM, 40 nM, 80 nM respectively (in all the other figures). Zn^2+^ concentrations were calculated with MaxChelator. Pipette solution contained (mM): 145 CsCl, 5 EGTA, 2.4 CaCl_2_ (~100 nM free Ca^2+^), 2 MgATP, and 10 HEPES, pH 7.4 with CsOH. For cell-attached recordings, bath KCl was substituted for NaCl to zero the membrane potential, and the pipette solution was (mM): 150 CsCl, 10 HEPES, 0.5 EGTA. Ion Selectivity Measurements were performed as previously described^14^. Briefly, the currents in the presence of varying external cations were measured upon rapidly exchanging the extracellular solution using a linear array of microperfusion pipes. The ion permeability relative to Cs^+^ was calculated from the reversal potential under bi-ionic conditions according to the following equations:

$$\begin{array}{rcl}\frac{{P}_{{\rm{X}}}}{{P}_{{\rm{Cs}}+}} & = & \exp ({E}_{{\rm{rev}}}\times \frac{F}{RT})\\ \frac{{P}_{{\rm{Ca}}2+}}{{P}_{{\rm{Cs}}+}} & = & \frac{{[{{\rm{Cs}}}^{+}]}_{i}}{4{[{{\rm{Ca}}}^{2+}]}_{o}}\times \exp ({E}_{{\rm{rev}}}\times \frac{F}{RT})\times (\exp ({E}_{{\rm{rev}}}\times \frac{F}{RT})+1)\end{array}$$

where Px/P_Cs+_ and P_Ca2+_/P_Cs+_ are the monovalent cation and Ca^2+^ permeability relative to Cs^+^, respectively, *E*_rev_ is the reversal potential, *F* is Faraday’s constant, *R* is the universal gas constant, and *T* is the absolute temperature. *E*_rev_ was corrected for the liquid junction potentials measured under the conditions of our experiments.

### Molecular modeling

Conformational modeling of the D915H tetramer complex with Zn^2+^ was performed using extensive energy-based sampling of the TRPA1 loop residues (910 to 920) in ICM-Pro (v3.8-7) molecular modeling software (molsoft.com). More than 10^6^ biased probability monte-carlo minimization steps were performed, with His915 isomerization state recalculated at each step.

### Chemicals

A-967079 and polygodial were purchased from Tocris (Bristol, United Kingdom). All the other chemicals (including cinnamaldehyde, HC-030031) were purchased from Sigma (St. Louis, MO). For stock solutions, cinnamaldehyde, HC-030031, A-967079 and 4,4′-Diisothiocyanato-2,2′-stilbenedisulfonic acid (DIDS) were dissolved in dimethyl sulfoxide (DMSO). Prior to use, the stock solution was diluted in the appropriate bath solution. The final dimethyl sulfoxide concentration was <0.5%.

## Electronic supplementary material

Supplementary Figures

## Data Availability

All data will be deposited in the relevant archive or available upon request. cDNA constructs will be submitted to Addgene or available upon request.
