# Supplementary material for: Activation Stoichiometry and Pore Architecture of TRPA1 Probed with Channel Concatemers
Source: Sci Rep. 2018 Nov 20;8:17104. doi: 10.1038/s41598-018-35435-y (PMC6244152; doi:10.1038/s41598-018-35435-y)
Supplement: Supplementary file 1 — Supplementary Figures [file 41598_2018_35435_MOESM1_ESM.pdf]

Supplementary Information

Activation Stoichiometry and Pore Architecture of TRPA1 Probed with Channel Concatemers

Wenlei Ye[1,2], Yu-Hsiang Tu[1], Alexander J. Cooper[1,3], Zheng Zhang[1], Vsevolod Katritch[1,4], Emily R. Liman[1]

[1] Department of Biological Sciences, University of Southern California, Los Angeles, CA 90089
[2] Present address: Department of Physiology, University of California, San Francisco, CA 94158, USA
[3] Present address: Zilkha Neurogenetics Institute, University of Southern California, Los Angeles, CA 90033
[4] Department of Chemistry, University of Southern California, Los Angeles, CA 90089

Correspondence:
Emily R. Liman
Section of Neurobiology
University of Southern California
3641 Watt Way
Los Angeles CA, 90089
Tel: 213-821-1454
liman@usc.edu

Wenlei Ye
Department of Physiology
University of California, San Francisco
1550 4th street San Francisco , CA, 94518
Wenlei.ye@ucsf.edu

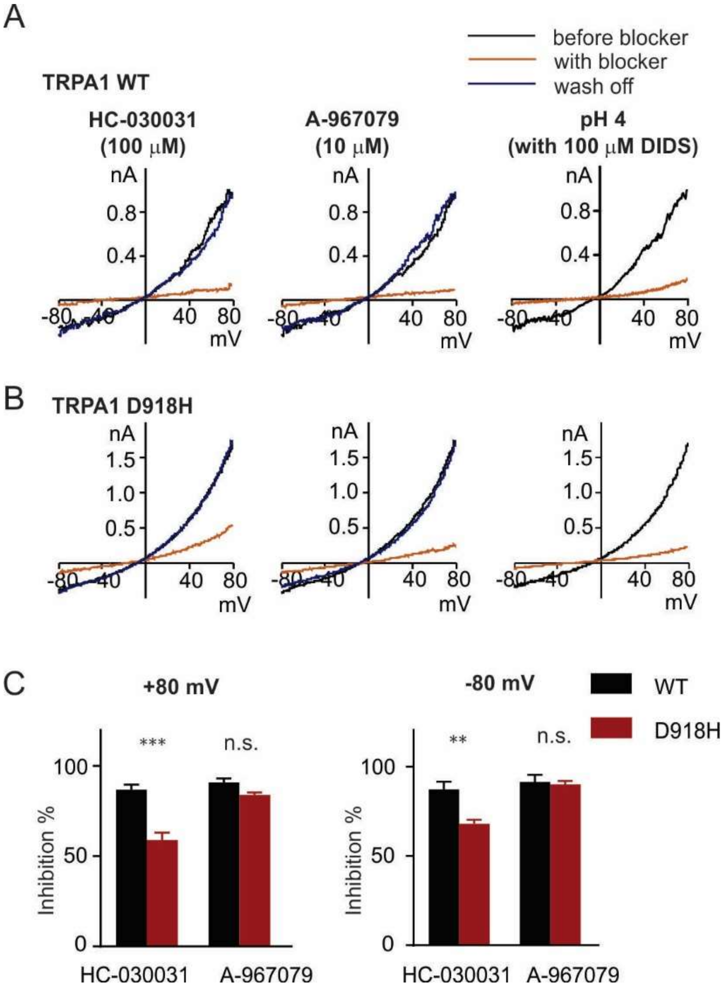

Figure S1

D918H mutation reduces the inhibition efficiency of HC-030031 but not of A-967079. (A, B) The I-V relationships of wild type (A) and D918H (B) TRPA1 currents evoked in response to cinnamaldehyde. The stimulus protocol was the same as in Fig. 1. The pH 4 solution contains 100 μM DIDS to avoid activation of acid-induced Cl- currents endogenous to HEK-293 cells. (C) Summary of inhibition percentage of the currents by each antagonist measured at +80 mV and -80 mV. n.s. $P > 0.05$, $^{**}P < 0.01$, $^{***}P < 0.001$ (Sidak's multiple comparison following two-way ANOVA); $N \geq 4$. Due to the contamination by HEK-cell endogenous acid-evoked Cl^- currents, acid inhibition was not quantified.

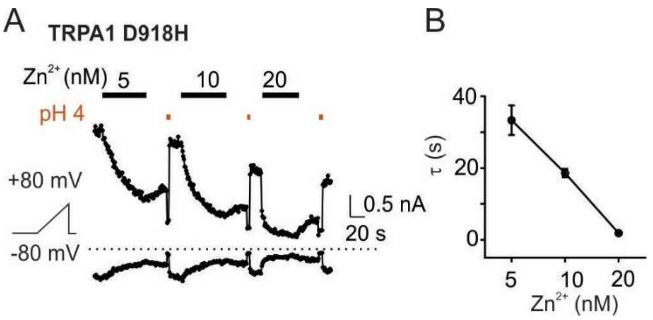

Figure S2

TRPA1 D918H inhibition by Zn^{2+} is irreversible. (A) Time course of the inhibition of TRPA1 D918H currents by Zn^{2+}. Solution at pH 4 was used to restore the current after wash-off of Zn^{2+}. (B) The average time constant (τ) of inhibition under indicated Zn^{2+} conditions. The time constant was obtained from a fit with a one-site binding model. $N \geq 3$. Note that 20 nM Zn^{2+} was able to block completely within a few seconds, consistent with Fig. 1D.

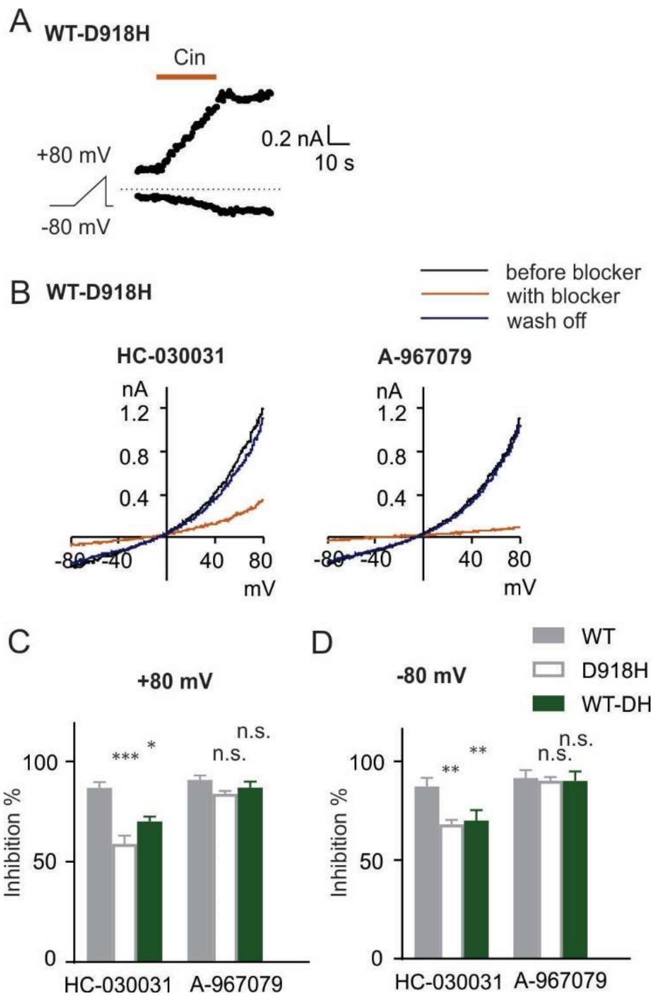

Figure S3

The dimer, WT-D918H, has a reduced sensitivity to HC-030031 but not A-967079. (A) Time course of WT-D918H currents activated in response to 100 µM cinnamaldehyde (Cin). (B) The I-V relationships of TRPA1 WT-D918H with and without the inhibitors, 100 µM HC-030031 and 10 µM A-967079. (C, D) Average inhibition percentage by the indicated antagonists measured at +80 mV and -80 mV. WT and D918H data were replotted from Fig. S1. n.s. $P > 0.05$, *$P < 0.05$, **$P < 0.01$, ***$P < 0.001$ (Sidak's multiple comparison against wild type following two-way ANOVA); $N \geq 4$.

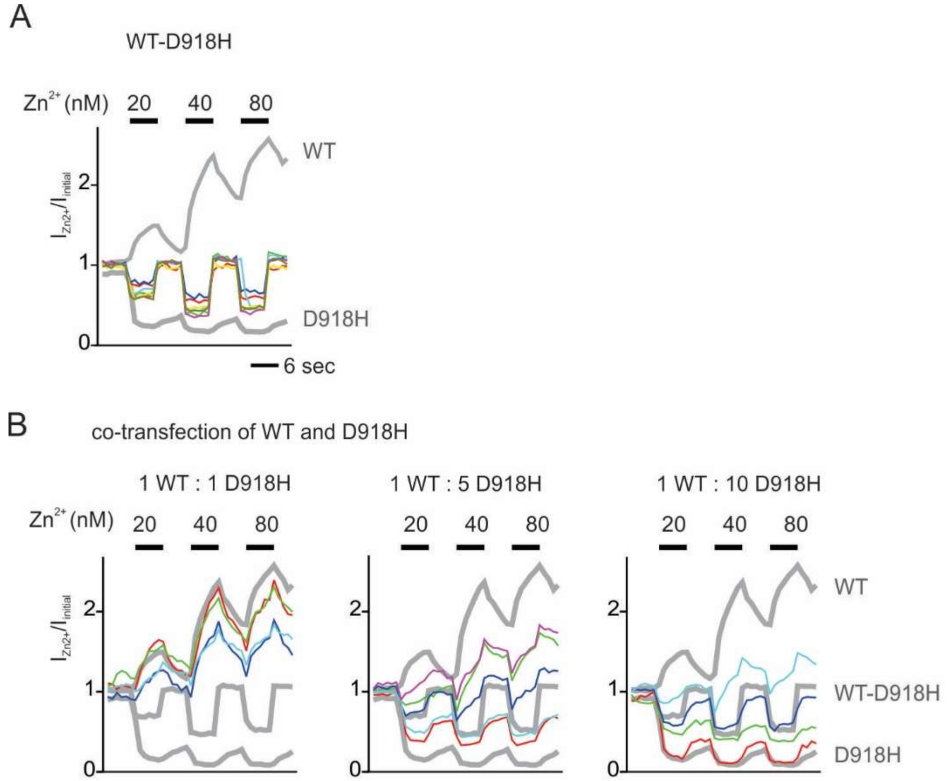

Figure S4

The dimer WT-D918H generates TRPA1 channels with a defined stoichiometry. Time course of normalized current responses to indicated Zn^{2+} concentrations in cells transfected with WT-D918H (A), or co-transfected with WT and D918H at indicated ratios (B). Each coloured trace represents one cell, while the upper, middle and lower shaded traces show the average response of WT, WT-D918H and D918H currents respectively. The experiment protocol was the same as in Fig. 1. Y-axis is the current magnitude normalized to the initial magnitude.

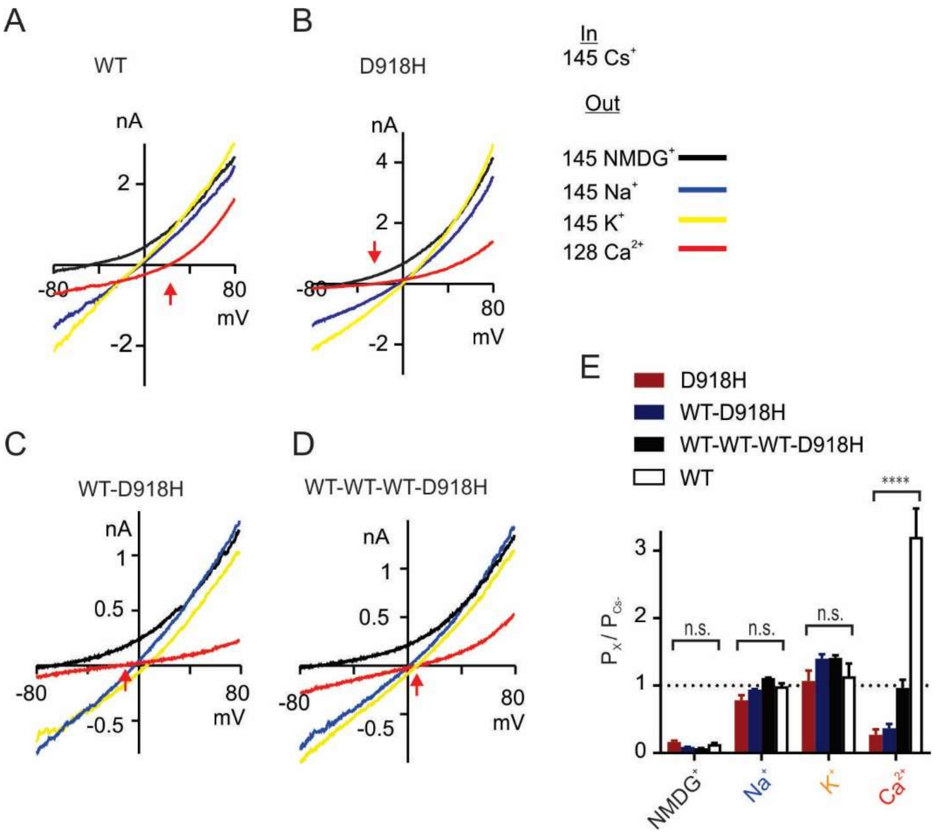

Figure S5

The Ca^{2+} selectivity is progressively reduced as the number of D918H subunits in the tetramer is increased. (A~D), the I-V relationships of indicated channels under bi-ionic conditions. (E) Summary of ion selectivities (P_X/P_{Cs+}) of the four vectors calculated as described in the methods. n.s. $P > 0.05$, ****$P < 0.0001$ (one-way ANOVA); $N \geq 3$. For P_{Ca2+}/P_{Cs+}, the subsequent Dunnett's multiple comparison test indicated the significant differences of D918H, WT-D918H, WT-WT-WT-D918H each against WT reached $P < 0.0001$.

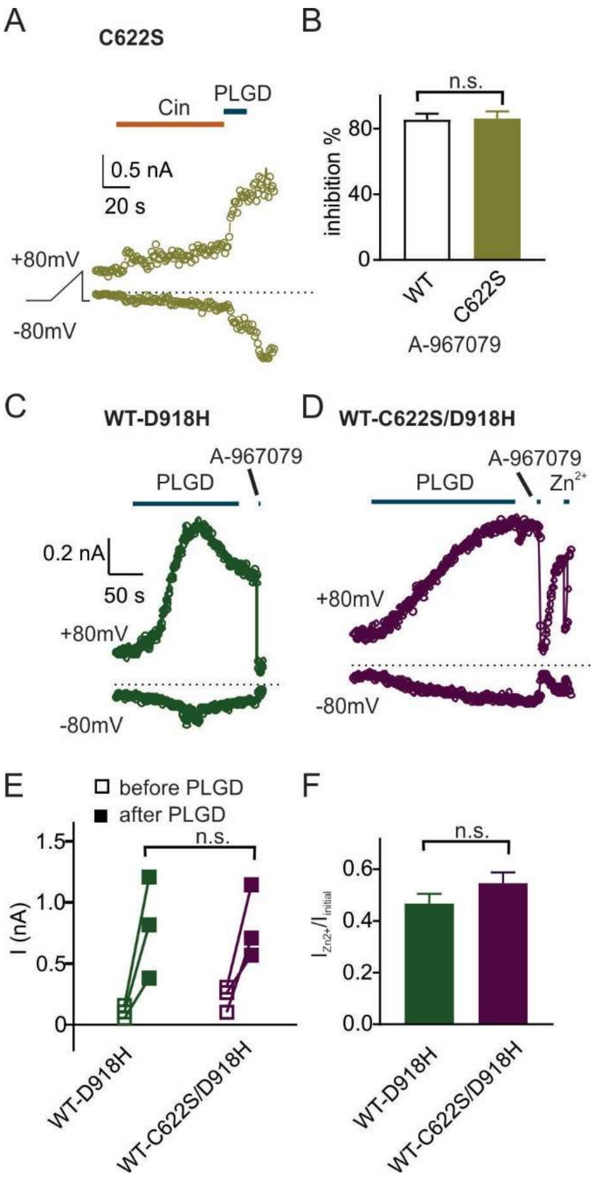

Figure S6

WT-D918H and WT-C622S/D918H are activated by polygodial. (A) Time course of TRPA1 C622S response to 100 μM cinnamaldehyde and 10 μM polygodial (PLGD). (B) Summary of inhibition percentage of PLGD-evoked currents by 10 μM A-967079; $N = 5\sim7$. n.s. $P > 0.05$ (unpaired *t*-test). (C, D) Time courses of TRPA1 WT-D918H and WT-C622S/D918H responses to polygodial. (E) Summary of current magnitudes measured before and after cinnamaldehyde treatment (peak current if the current decays), n.s. $P > 0.05$ (two-way ANOVA). (F) Summary of current fractional change in response to 40 nM Zn^{2+}; $N = 3\sim4$. n.s. $P > 0.05$ (unpaired *t*-test)

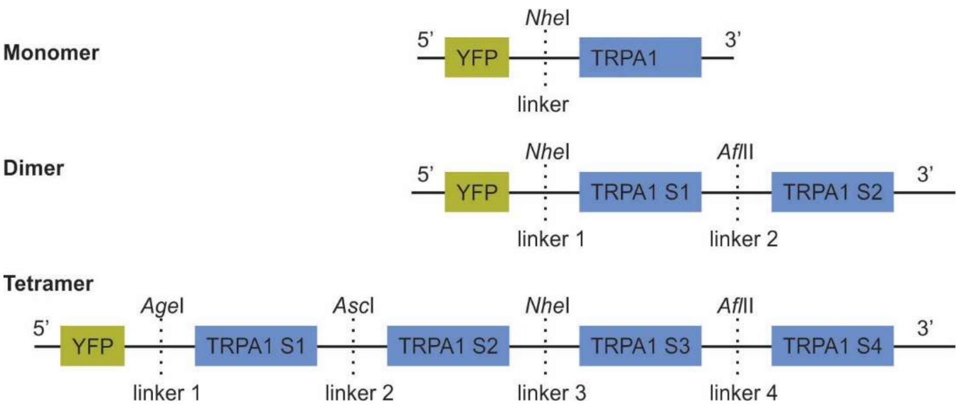

Figure S7

Composition of YFP-fused TRPA1 and TRPA1 concatemeric constructs. YFP cDNA was colored yellow and TRPA1 was colored blue. Restriction enzyme recognition sites were labeled with dash lines.
